# Congratulations, You’re Pregnant! Now About Your Shifts . . . : The State of Maternity Leave Attitudes and Culture in EM

**DOI:** 10.5811/westjem.2017.6.33843

**Published:** 2017-07-17

**Authors:** Casey Z. MacVane, Megan L. Fix, Tania D. Strout, Kate D. Zimmerman, Rebecca B. Bloch, Christine L. Hein

**Affiliations:** * Maine Medical Center, Department of Emergency Medicine, Portland, Maine; † Tufts University School of Medicine, Boston, Massachusetts; ‡ University of Utah Hospital, Department of Emergency Medicine, Salt Lake City, Utah

## Abstract

**Introduction:**

Increasing attention has been focused on parental leave, but little is known about early leave and parental experiences for male and female attending physicians. Our goal was to describe and quantify the parental leave experiences of a nationally representative sample of emergency physicians (EP).

**Methods:**

We conducted a web-based survey, distributed via emergency medicine professional organizations, discussion boards, and listservs, to address study objectives.

**Results:**

We analyzed data from 464 respondents; 56% were women. Most experienced childbirth while employed as an EP. Fifty-three percent of women and 60% of men reported working in a setting with a formal maternity leave policy; however, 36% of women and 18% of men reported dissatisfaction with these policies. Most reported that other group members cover maternity-related shift vacancies; a minority reported that pregnant partners work extra shifts prior to leave. Leave duration and compensation varied widely, ranging from no compensated leave (18%) to 12 or more weeks at 100% salary (7%). Supportive attitudes were reported during pregnancy (53%) and, to a lesser degree (43%), during leave. Policy improvement suggestions included the development of clear, formal policies; improving leave duration and compensation; adding paternity and adoption leave; providing support for physicians working extra to cover colleagues’ leave; and addressing breastfeeding issues.

**Conclusion:**

In this national sample of EPs, maternity leave policies varied widely. The duration and compensation during leave also had significant variation. Participants suggested formalizing policies, increasing leave duration and compensation, adding paternity leave, and changing the coverage for vacancies to relieve burden on physician colleagues.

## INTRODUCTION

The dramatically shifting demographics of medicine has been well documented in recent decades. Over the last 50 years, the number of women in medicine has climbed steadily. In 1970, fewer than 10% of medical students were female, while in 2013 47% of medical school matriculants were women.^1^ Women represented 38% of U.S. medical school faculty in 2013, up from 32% in 2003 and less than 10% in the 1970s.^2^ This trend has been noted within the specialty of emergency medicine (EM) as well, with a rise in female residents from 28% in 2001 to 38% in 2013.^3^ As gender balance has continued to evolve in medicine, issues related to childbearing – in particular to pregnancy, maternity leave, and early childhood care – have risen to the forefront.

Concurrent with the notably increased presence of women in medicine has been the growing number of physicians representing *Generation X*, those born between 1964 and 1980, and *Millennials,* also known as *Generation Y* (born between 1980 and 1999). Currently, Generation Xers make up 30% of practicing physicians.^4^ Millennials represent only 5% of practicing physicians, but will clearly play a growing role in the future.^4^ These two generational cohorts both tend to place increased value on work-life balance compared to prior generations.^5^–^6^ Maternity leave exemplifies an issue that is supremely important both to women and to physicians who highly value work-life balance.

Increasing awareness of physician burnout and the subsequent focus on physician wellness lends importance to the issue of maternity leave and parental leave in general. Traditionally, maternity leave has been more likely to be clearly defined and accepted in professions outside of medicine and in countries outside of the United States.^7^–^9^ However, with growing numbers of physicians beginning their careers who value family, career flexibility and wellness, these types of issues are likely to become a more important component of the medical landscape in the U.S. Moreover, effective maternity leave policies could become an important organizational strategy to address physician wellness.^6^

Despite the fact that maternity leave inevitably impacts many practicing physicians, little scientific literature on the issue exists. While multiple authors have addressed maternity leave as it affects resident physicians^10^–^11^ and specialties outside of EM,^13^–^14^ our review revealed only one EM-specific article relating to maternity leave.^15^ Lewin focused primarily on the dearth of policies relating to family leave during residency and suggested some parameters for an ideal policy. Further complicating the current discussion is the associated confusion between parental- and family-leave policy nomenclature. For the purposes of this article, maternity and paternity leave refer specifically to maternal and paternal time away from work due to pregnancy and delivery. Family leave, although beyond the scope of this discussion, refers to time away from work due to personal or family illness of various etiologies. Adoption leave policies are also considered distinctly from maternity leave. While there is clearly a paucity of data regarding maternity leave practices in EM, there is even less knowledge regarding how these issues affect male physicians.

To address these gaps, we sought to assess the composition of existing maternity leave policies, as well as to evaluate for differences in physicians’ experiences with maternity leave based upon gender and work setting. Additionally, we investigated the attitudes and opinions of a national sample of emergency physicians (EP), both male and female. Lastly, we asked for suggestions regarding how the current maternity leave landscape could be enhanced so that administrators and policy makers can continue to provide high job satisfaction given the changing demographic of EPs.

Population Health Research Capsule

What do we already know about this issue?

Despite implications for health, wellness, burnout prevention, and work-life balance, very little is known about the maternity leave experiences of attending emergency physicians.

What was the research question?

What are the experiences and attitudes regarding maternity leave for United States emergency physicians?

What was the major finding of the study?

Emergency physicians’ maternity leave experiences and beliefs varied widely and there were many suggestions to improve the current state of parental leave.

How does this improve population health?

Maternity leave policies may be an important wellness and burnout prevention strategy to ensure a robust emergency physician workforce is available to provide high-quality patient care.

## METHODS

### Study Design

We performed this descriptive study with the development and use of a web-based survey to collect data regarding attitudes and policies related to maternity leave for physicians in EM. The study was reviewed and determined to be exempt by the Maine Medical Center Institutional Review Board.

### Selection of Participants

Chapter executives from six state chapters of the American College of Emergency Physicians (ACEP) agreed to distribute the survey link to their memberships electronically using the web-based survey tool SurveyMonkey® (Survey Monkey, Palo Alto, CA). Participating state chapters included Maine, Massachusetts, Missouri, Ohio, Utah and Virginia. While we contacted all ACEP state chapters for inclusion in the study, ultimately six state chapters agreed to participate and comprised the final convenience study sample. In addition, the survey link was distributed via an electronic listserv for the American Association of Women Emergency Physicians (AAWEP). The survey was sent to all members of these professional organizations who provided electronic mail addresses to their membership offices.

### Methods and Measurements

The survey was created by the study investigators following a comprehensive review of the existing scientific literature and was designed to collect data with regard to four main topics of consideration: a) to identify maternity leave policies currently available to EPs, b) to determine the individual experience of pregnancy and maternity leave for EPs, c) to assess attitudes of colleagues and supervisors in relation to maternity leave, and d) suggestions to improve parental leave policies. Items pertaining to each of these main concepts were developed and revised for clarity after review by fellow EPs.

We developed two versions of the survey, one for men and one for women. Participants’ responses to initial demographic questions determined which of the two versions would be administered. The female survey included 42 questions while the male survey included 28 questions. For the female survey, we collected the following data: a) age, b) number of children, c) having children while employed as an EP, d) delivering or taking leave prior to 37 weeks gestation, e) structure of respondent’s work week, f) the average number of clinical hours per week, g) practice and group type (academic/community/other, private/hospital/other and gender ratio), and h) whether the respondent was the primary source of income for her family.

For the male survey, we collected the following data: a) age, b) number of children, c) having children while employed as an EP, d) whether a female colleague had a child while the respondent was employed as an EP, e) structure of respondent’s work week, f) the average number of clinical hours per week, g) practice and group type (academic/community/other, private/hospital/other and gender ratio), and h) whether the respondent was the primary source of income for his family.

Both the female and male versions of the survey included specific questions regarding the respondent’s current maternity leave policies, as well as their attitudes towards these policies and their attitudes towards colleagues who have taken maternity leave.

### Data Collection

We collected data anonymously using a modified version of Dillman’s approach.^16^ A series of three electronic mail messages was distributed with the survey link to potential participants over a period of approximately eight weeks in 2011. These included an initial message explaining the study and including the survey link, a reminder message two weeks later, and a final reminder e-mail one week after that. Responses to the survey were accepted for several more weeks following the final electronic message.

Study participants received an initial message from their professional organization that explained the objective of the study and included a link to the survey. Upon opening the web-based survey, an informational page further explained the study objectives and reviewed the voluntary, anonymous nature of the study. No identifiers were collected. We excluded potential study subjects if they were still in residency or were medical students. Surveys that were opened and submitted without responses were also excluded from analysis.

### Statistical Analysis

We downloaded raw study data from the SurveyMonkey® website into a Microsoft Excel (Microsoft, Inc., Redmond, WA) spreadsheet. Quantitative analysis was completed using SPSS version 22 (SPSS, Inc., Chicago, IL) statistical software. We described categorical data with numbers and percentages. Qualitative responses were evaluated for common themes, which were organized into thematic categories.

## RESULTS

### Characteristics of the Study Participants

A total of 530 participants opened the survey link, provided informed consent, and completed at least part of the study survey. We excluded 66 participants (64 resident physicians and two medical students), leaving data from 464 responding attending physicians for analysis. The study sample was comprised of 256 (56%) women and 204 (44%) men; respondents most frequently reported being between 30 and 39 years old (42%, n = 189) and having two children (35%, n = 158). The majority of respondents experienced the birth of at least one child while employed as an EP, including 65% of women (n = 163) and 70% of men (n = 138). Sixty-eight percent of women (n = 166) and 91% of men (n = 180) reported being the primary earner for their household, with 78% of women (n = 187) and 92% of men (n = 183) reporting working full time. Distinctions between full-time and part-time work status were left to the discretion of the study respondents. Regarding work settings, female and male respondents most frequently reported being a hospital employee (44% and 37% respectively), and both female and male respondents reported working in a community setting most often (39% and 56% respectively). Additional detail regarding the characteristics of the study subjects is available in the Table.

### Maternity Leave Policies

**Figure 1:**
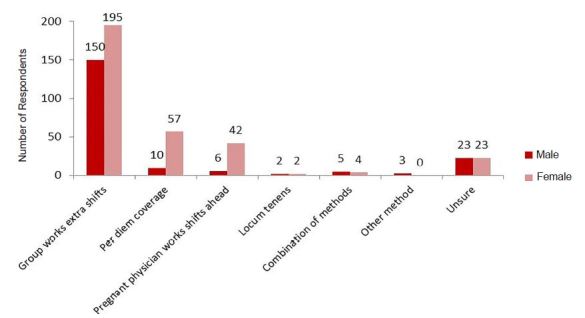
Methods for covering shift vacancies as reported by male and female respondents in survey regarding parental leave policies.

Fifty-three percent of women (n = 129) and 60% of men (n = 119) reported working in a setting with a formal maternity leave policy; however 36% of women (n = 82) and 18% of men (n = 35) reported dissatisfaction with those policies. Seventeen percent of women and 21% of men were unaware of whether there was a formal maternity leave policy at their current place of employment. Most respondents reported that vacant shifts created by maternity leave were covered by other group members working extra shifts (76% female, n = 191; 75% male, n = 149), with a minority reporting that pregnant partners work extra shifts prior to maternity leave (17% female, n = 42; 10% male, n = 19). Maternity leave duration and compensation varied widely, ranging from no compensated leave (18%, n = 21) to 12 or more weeks at 100% salary (8%, n = 8). Many participants reported needing to use paid time off (23%, n = 53) or vacation time (43%, n = 99) to cover their maternity leave, and others (13%, n = 13) cited the Family Medical Leave Act as the basis for their maternity leave policy. Twenty-one percent of respondents (n = 26) believed that their institution’s maternity leave policy had been implemented or reviewed within the prior five years, while 65% (n = 79) were unsure in this regard. Payback of shifts missed during maternity leave was reported as being required by 10% (n = 23) of women. Additionally, survey respondents also reported that working extra shifts in advance of maternity leave was not an option 14% of the time (n = 31). Figure 1 provides information regarding the manner in which participants reported that vacant shifts are covered in their departments.

### Individual Experiences of Pregnancy and Maternity Leave for Emergency Physicians

**Figure 2:**
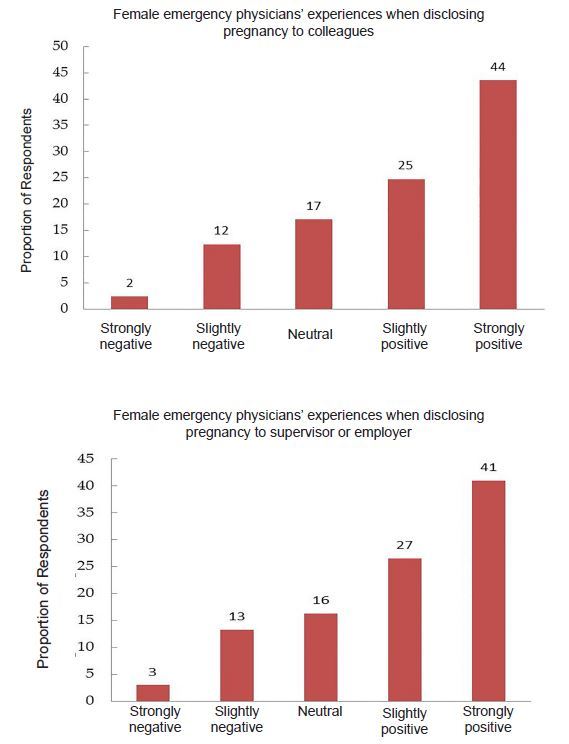
Emergency physician experiences with pregnancy disclosure.

**Figure 3:**
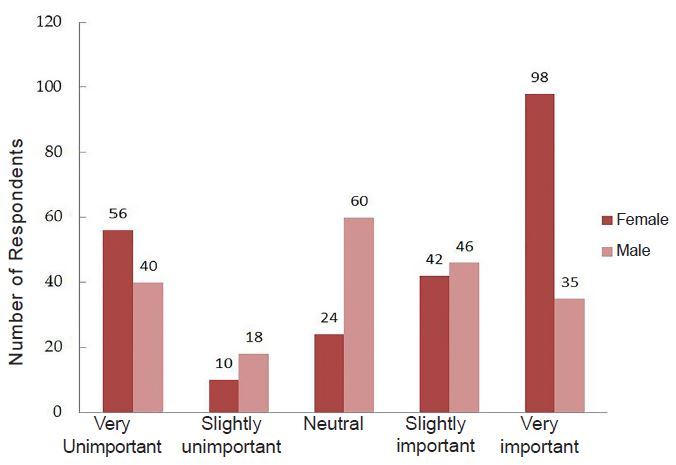
Importance of maternity leave policies for emergency physicians.

Forty-six percent of female participants reported feeling guilt or other negative emotions during their maternity leave. In particular, the experience of pregnant EPs and perceived support of colleagues and supervisors varied significantly when disclosing their pregnancy (Figure 2). Eight percent of women reported considering leaving a job due to maternity leave policies and 17% delayed pregnancy due to leave policies. While 61% of women reported that maternity leave policies are slightly to very important to them, 41% of men reported the same. (Figure 3).

### Emergency Physician’s Beliefs and Attitudes

**Figure 4:**
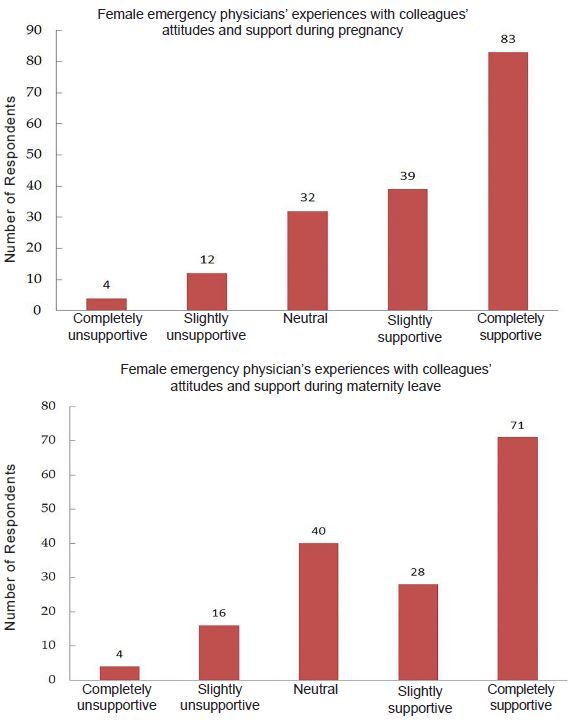
Emergency physicians’ experiences regarding colleagues’ attitudes and support.

Fifty-three percent of women reported supportive attitudes from colleagues during pregnancy, and 43% reported supportive attitudes during maternity leave (Figure 4). Seventy percent of participants worked extra shifts for a colleague during her maternity leave with 80% of those respondents reporting neutral or positive attitudes about covering the vacancy. The majority of survey participants (78%) reported slightly supportive to very supportive attitudes during colleagues’ pregnancies. This is consistent with the fact that 71% of participants rated their level of supportiveness during colleagues’ maternity leaves as slightly to very supportive.

### Suggestions to Improve Parental Leave Policies

**Figure 5:**
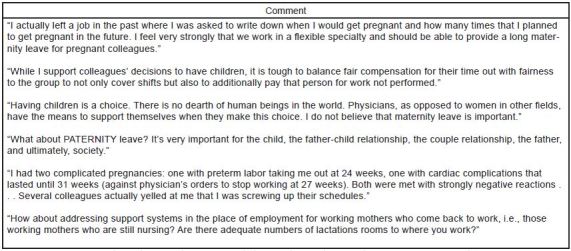
Examples of participant comments in response to the question, “Is there anything else you would like to add?”

Almost uniformly, respondents recognized the importance of establishing clear and formal maternity leave policies so that female EPs would have realistic expectations of their pregnancy and maternity leave. Another common suggestion was the need for clear paternity leave policies as well. Other recommendations included increasing parental leave duration and compensation; adding a component for adoption; adding support for those physicians working extra shifts to cover colleagues’ leave; and addressing breastfeeding issues for women returning to work. Representative comments are included in Figure 5.

## DISCUSSION

Parental leave continues to gain national attention. During the first White House Summit on Working Families, President Obama highlighted that the United States is the only developed country that does not offer paid maternity leave.^7^ In medicine and particularly EM, a specialty that thrives on changing schedules, the way to accomplish paid parental leave is unclear and potentially more challenging.

This study represents the first nationally representative survey to our knowledge of male and female EPs regarding the subject of maternity leave policies. We addressed maternity leave policies, including individual experiences with current policies, beliefs and attitudes of EPs and suggestions for improved parental leave policies. About half of our respondents work in a setting with a formal leave policy. Many of these physicians, however, are dissatisfied with their policies. Maternity leave duration and compensation varied widely in our sample – ranging from no compensated leave to 12 or more weeks at 100% salary. One curious finding from our study was a difference in perceived attitudes. Most physicians reported that they have worked extra shifts for colleagues on leave and the majority of those reported neutral or positive attitudes about covering the vacancy. This is contradictory to the perceived attitudes of physicians who have taken maternity leave where only half of women reported supportive attitudes from colleagues during pregnancy and maternity leave. It is unclear why this discrepancy exists; however, the development of formal policies may help women feel more supported during leave when following agreed-upon hospital or departmental policies.

Overall, we found that maternity leave is an important topic to both those taking and covering for leave. In fact, some women reported considering leaving a job due to a maternity leave policy. These findings are reflected in multiple other studies, which have also demonstrated the importance of parental leave policies to practicing physicians.^17^–^19^ Our respondents suggested improvements in policies including enhancing leave duration and compensation; adding a component for adoption; adding support for those working extra shifts to cover colleagues’ leave; and addressing breastfeeding issues for women returning to work.

Our respondents reported variability in the presence, length and compensation for maternity leave. Although literature is scarce covering parental leave policies, it echoes our findings of inconsistency.^19^ This lack of consistency suggests the need for clear, formal policies. While there is little data on how to establish parental leave policies, some essential components include involving key hospital administrators as well as physicians and maintaining a focus on the financial implications for both.

Compensation for family leave also varies, and in 18% of our study sample there was no compensation for family leave at all. This compensation, however, can be an important source of employee satisfaction. A survey of over 1,300 female EPs investigated career satisfaction. They found that important personal predictors of satisfaction were schedule flexibility, supportive colleagues, and fairness of financial compensation.^20^

While we did not set out to look specifically at paternity leave policies, this topic was mentioned by numerous respondents. In concert with this, there has been a recent rise in awareness of paternity leave policies and we would be remiss not to mention their importance. Companies such as Yahoo are acknowledging the importance of paternity leave by offering fathers eight weeks of paid parental leave. This kind of policy is rare in the U.S. where only 13% of employers offer any paid paternity leave.^21^ The absence of clear paternity leave policies places the U.S. far behind other countries where paternity leave is an accepted and established practice. For example, in Sweden 85% of fathers take parental leave.^22^ Even for physicians, it is more common for men in other countries to take paternity leave. In England, 50–96% of male physicians take paternity leave.^23^ This demonstrates that it is possible for paternity leave to be accepted and supported.

Another prominent concern in our survey was shift coverage for vacancies during an EP’s maternity leave. This was covered by a variety of methods. The majority of respondents reported that coworkers worked extra shifts to fill these vacancies. Other methods included additional per diem staff coverage and working a heavier shift load prior to maternity leave. Although there is no single correct means for covering shifts, it is clear that coverage for parental leave in a fair and uniform means is an essential adjunct to the implementation of a successful maternity leave policy.

## LIMITATIONS

Our study has several limitations to consider. As with most surveys, there may be a tendency of those with strong opinions to participate, which may bias the responses. Additionally, this study is limited by the fact that our data collection was a convenience sample of state chapters of ACEP. We surveyed six state chapters, selected due to the fact that these were the states whose administrators responded to our original e-mail requesting distribution of our survey. Although these six chapters (Maine, Massachusetts, Missouri, Ohio, Utah and Virginia) were ultimately quite diverse in geographic location, it is possible that our results are biased due to the inability to survey all state chapters. We also included AAWEP, which due to its mission and membership may have biased the sample towards those concerned about maternity leave issues. It should be noted that there were more participants who reported being involved with academic practice, at least to some degree, and therefore our findings may not fully represent the perspectives of community-based EPs. Another potential limitation of our study is that we asked respondents about the maternity leave policies at their current place of employment, as opposed to the site of their practice when they had children. We did this with the intention of surveying the current state of maternity leave policies, but it is possible that we missed historical perspectives on maternity leave that may have informed the respondents’ current opinions.

Our inability to calculate an accurate response rate may limit the generalizability of our results. State chapter executives were unable to accurately report the total number of chapter members, so our study participants represented an unknown percentage of each chapter’s membership.

The final limitation of our study is due to its initial design. We were unable to use a formally validated survey instrument as none were available in the literature. We therefore designed our survey independently, based upon questions and topics raised during an extensive literature review. It is possible, however, that our questions did not fully explore all the intended content areas regarding attitudes and policies related to maternity leave for physicians in EM.

## CONCLUSION

Given the changing environment of our workforce and the generation shift to more Millennials, establishing formal parental leave policies will only become more important. We expect that as beliefs and attitudes continue to shift, this issue will become more important and will look differently, perhaps bringing more attention to associated concerns like paternity leave. Our study of male and female EPs found that the number of formal policies, satisfaction with leave policies, duration of and compensation for leave, as well as physician attitudes surrounding leave, vary considerably. Future research and efforts should focus on establishing guidelines for formal parental leave policies in EM. Along with the respondents in our study, we suggest that future policies include consistent and improved leave duration and compensation, paternity leave, adoption leave, and potentially address breastfeeding. Improvements in these policies will benefit not only physicians taking parental leave, but also have a significantly positive impact on colleagues, EM practice groups and the culture of emergency medicine as a whole.

## Figures and Tables

**Table:** Characteristics of emergency physician participants in a survey regarding maternity leave policies.

Characteristic	Female n = 256	Male n = 204
Age range, n (%)		
20–29 years	2 (0.8)	1 (0.5)
30–39 years	132 (52.4)	57 (28.6)
40–49 years	65 (25.8)	65 (32.7)
50–59 years	42 (16.7)	47 (23.6)
60–69 years	10 (4.0)	25 (12.6)
70–79 years	1 (0.4)	3 (1.5)
Primary household earner, n (%)		
Yes	166 (68.3)	180 (91.4)
No	77 (31.7)	17 (8.6)
Number of children, n (%)		
None	60 (23.7)	24 (12.1)
Currently pregnant	4 (1.6)	0 (0)
One	57 (22.5)	25 (12.6)
Two	89 (35.2)	69 (34.8)
Three	36 (14.2)	51 (25.8)
Four	4 (1.6)	18 (9.1)
Five	2 (0.8)	9 (4.5)
Six	1 (0.4)	1 (0.5)
Seven	0 (0)	1 (0.5)
Had child while in EM, n (%)		
Yes	163 (64.7)	138 (69.7)
No	89 (35.3)	60 (30.3)
Workweek structure, n (%)		
Full time	187 (77.6)	183 (92.0)
Part time	50 (20.7)	14 (7.0)
Per diem	4 (1.7)	2 (1.0)
Clinical hours per week		
None	6 (2.5)	2 (1.0)
< 20 hours	24 (10.0)	15 (7.6)
20–29 Hours	76 (31.5)	44 (22.3)
30–39 Hours	97 (40.2)	89 (45.2)
40–49 Hours	34 (14.1)	38 (19.3)
≥ 50 hours	4 (1.7)	9 (4.6)
Years of EM practice, n (%)		
None	2 (0.8)	1 (0.5)
>1 – 5 Years	98 (40.1)	47 (23.6)
6 – 9 Years	47 (19.5)	25 (12.6)
10 – 15 Years	42 (17.4)	34 (17.1)
16 – 19 Years	9 (3.7)	20 (10.1)
20 – 25 Years	28 (11.6)	28 (14.1)
26 – 30 Years	11 (4.6)	23 (11.6)
>30 Years	4 (1.7)	23 (11.6)
Primary employer type, n (%)		
Academic practice	86 (35.4)	45 (22.7)
Community practice	97 (39.9)	110 (55.6)
Community/academic	55 (22.6)	40 (20.2)
Other	5 (2.1)	3 (1.5)
Group structure, n (%)		
Contract group	74 (30.5)	62 (31.1)
Hospital employee	108 (44.4)	74 (37.2)
Private practice	39 (16.0)	49 (24.6)
Other	22 (9.1)	14 (7.0)
Percent female in group, n (%)		
None	0 (0)	5 (2.6)
<5% Female	3 (1.3)	0 (0)
5 – 10% Female	23 (9.9)	30 (15.3)
11 – 20% Female	55 (23.6)	50 (25.5)
21 – 30% Female	63 (27.0)	48 (24.5)
31 – 40% Female	41 (17.6)	36 (18.4)
41 – 50% Female	32 (13.7)	23 (11.7)
51 – 60% Female	8 (3.4)	1 (0.5)
61 – 70% Female	3 (1.3)	0 (0)
71 – 80% Female	3 (1.3)	0 (0)
81 – 90% Female	0 (0)	0 (0)
91 – 100% Female	1 (0.4)	0 (0)
Unsure	1 (0.4)	3 (1.5)

*EM*, emergency medicine.
